# High expression of astrocyte elevated gene-1 (AEG-1) is associated with progression of cervical intraepithelial neoplasia and unfavorable prognosis in cervical cancer

**DOI:** 10.1186/1477-7819-11-297

**Published:** 2013-11-20

**Authors:** Ke Huang, Li An Li, Yuanguang Meng, Yanqin You, Xiaoyu Fu, Lei Song

**Affiliations:** 1 Department of Obstetrics and Gynecology, Chinese PLA General Hospital, 28 Fuxing Road, Beijing 100853, China

**Keywords:** AEG-1, Cervical cancer, Tissue microarrays, Prognosis

## Abstract

**Background:**

Astrocyte elevated gene-1(AEG-1) plays an important role in the development and progression of certain types of human cancers. However, the expression dynamics of AEG-1 in cervical cancer and its clinical/prognostic significance are unclear.

**Method:**

In present study, the methods of tissue microarrays (TMA) and immunohistochemistry (IHC) were utilized to investigate AEG-1 expression in cervical intraepithelial neoplasia (CIN) and cervical cancer. Receiver operating characteristic (ROC) curve analysis, *χ*2 test, Kaplan-Meier plots, and multivariate Cox regression analysis were used to analyze the data.

**Results:**

The expression level of AEG-1 was increased from CIN I to CIN III. High expression of AEG-1 could be observed in 61.1% (55/90) of cervical cancer. Moreover, high expression of AEG-1 correlated with tumor size and lymph node metastasis (all *P* <0.05). More importantly, high expression of AEG-1 was closely associated with cervical cancer patient shortened survival time as evidenced by univariate and multivariate analysis (*P* <0.05).

**Conclusions:**

Our data suggest for the first time that high expression of AEG-1 is associated significantly with progression of cervical cancer. AEG-1 overexpression, as examined by IHC, has the potential to be used as an immunomarker to predict prognosis of cervical cancer patients.

## Background

Cervical cancer is the second most frequent cancer in women. Cervical lesions advance from cervical intraepithelial neoplasia (CIN) to cervical cancer is a complicated process initiated by persistent infection with high-risk human papillomavirus (HPV) [1]. Currently, therapies include operation combined with chemotherapy and radiotherapy are major treatment means of the cervical cancer. However, these treatment methods are effective only in limited cases. Therefore, it is of great value to better understanding of molecular events and prognostic markers as well as novel therapeutic strategies of cervical cancer.

Astrocyte elevated gene-1(AEG-1) was originally identified as a human immunodeficiency virus-1 (HIV-1)-inducible gene in human fetal astrocytes [2]. Recently, numerous studies have shown the key role of AEG-1 in the development and progression of cancer [3,4]. Aberrant elevation of AEG-1 expression occurs in a series of human cancers, such as gastric cancer, breast cancer, osteosarcoma, non-small cell lung cancer, and colon cancer, compared to the matched non-neoplastic regions [5-9]. AEG-1 was found to promote the proliferation of breast cancer cells by downregulating FOXO1 through the PI3K/AKT signaling pathway [10]. Interestingly, in HeLa cells, overexpression of AEG-1 significantly enhances the invasive ability of cells, while inhibition of AEG-1-induced NF-κB decreases invasion [11]. Furthermore, AEG-1 has been found to upregulate MMP-9 and induce human glioma cell invasion [12]. These reports suggest that the AEG-1 might be involved in the progression of multiple cancers.

However, there are few reports concerning the association between AEG-1 expression and progression of cervical intraepithelial neoplasia or patient survival in cervical cancer. Therefore, in the present study, we evaluated AEG-1 protein expression in CIN and cervical cancer patient samples to investigate its role in cervical lesions, and its correlation with clinicopathological variables.

## Methods

### Patients and tissue specimens

A total of 90 unselected cervical cancer samples were collected between 1999 and 2001 from the Department of Pathology, Chinese PLA General Hospital. The tumor cases included 74 cases of squamous carcinoma and 16 cases of adenocarcinoma. Moreover, cases of CIN I, CIN II, and CIN III were 18, 17, and 15 respectively. Hysteromyoma as normal control cervical tissues (*n* = 10) that obtained from surgically removed uteruses. Ages of the 90 patients with cervical cancer ranged from 30 to 65 years (mean, 48.1 years). Clinicopathological characteristics of the tumor cases are presented in Table 1. The Institute Research Medical Ethics Committee of Chinese PLA General Hospital granted approval for this study.

**Table 1:** Relationship between AEG-1 expression and clinical parameters in cervical cancer patients

**Clinical parameter**	**Case**	**AEG-1 expression**		***P*** **value**
		**Low**	**High**	
Age (years)				
<50	35	15	20	
≥50	55	20	35	0.658
Tumor size (cm)				
<4	63	30	33	
≥4	27	5	22	0.010^a^
Histological classification				
Squamous carcinoma	74	26	48	
Adenocarcinoma	16	9	7	0.158
Clinical stage				
I + II	53	17	36	
III + IV	37	18	19	0.129
Pathological grade				
G_1_	19	10	9	
G_2+_ G_3_	71	25	46	0.192
Lymph node metastasis				
Absent	52	27	25	
Present	38	8	30	0.004^a^

^a^ < 0.05.

### Tissue microarrays (TMA) construction and immunohistochemistry

Tissue microarray was constructed as the method described previously [13]. Briefly, the paraffin-embedded tissue blocks and the corresponding histological H&E-stained slides were overlaid for tissue TMA sample. Triplicate 0.6 mm diameter cylinders were punched from representative areas of an individual donor tissue block, and re-embedded into a recipient paraffin block at a defined position, using a tissue arraying instrument (Beecher Instruments, Silver Spring, MD, USA).

The TMA blocks were cut into 4-μm sections and deparaffinized by routine techniques. First, the slides were microwaved in 10 mM citrate buffer (pH 6.0) for 10 min. Then the slides were incubated with anti-AEG-1 (ab76742, 1:100 dilution, Abcam, UK) and stored overnight at 4°C. The slides were sequentially incubated with a secondary antibody (Maxim-Bio, Fuzhou, China). Labeling was detected by adding diaminobenzidine (Maxim-Bio). Sections were then counterstained with hematoxylin. A negative control was obtained by replacing the primary antibody with a normal murine IgG.

### Evaluation by immunohistochemistry

Immunoreactivity for AEG-1 protein was scored using a semi-quantitative method by evaluating the number of positive tumor cells over the total number of tumor cells. Scores were assigned by using 5% increments (0%, 5%, 10%,… 100%). Expression for AEG-1 was assessed by two independent pathologists.

### Selection of cutoff scores

Receiver operating characteristic (ROC) curve analysis was performed to determine cutoff score for tumor ‘high expression’ by using the 0,1-criterion [14]. At AEG-1 score, the sensitivity and specificity for each outcome under study was plotted, thus generating various ROC curves. The score was selected as the cutoff value which was closest to the point with both maximum sensitivity and specificity. Tumors designated as low expression of AEG-1 were those with the scores below or equal to the cutoff value, while tumors of high expression were those with scores above the value. To use ROC curve analysis, the clinicopathologic characteristics were dichotomized: tumor size (<4 cm *vs.* ≥4 cm), pathological grade (G_1_*vs.* G_2_ + G_3_), clinical stage (I + II *vs.* III + IV), lymph node metastasis (Yes *vs.* No).

### Statistical analysis

Statistical analysis was performed by using the SPSS statistical software package (standard version 17.0; SPSS, Chicago, IL, USA). ROC curve analysis was applied to determine the cutoff score for high expression of AEG-1. The associations between AEG-1 expression and other variables were analyzed by using *χ*2 test. For univariate survival analysis, survival curves were obtained with the Kaplan-Meier method. *P* <0.05 denotes the presence of a statistically significant difference.

## Results

### AEG-1 is upregulated from CIN I to CIN III

**Figure 1:**
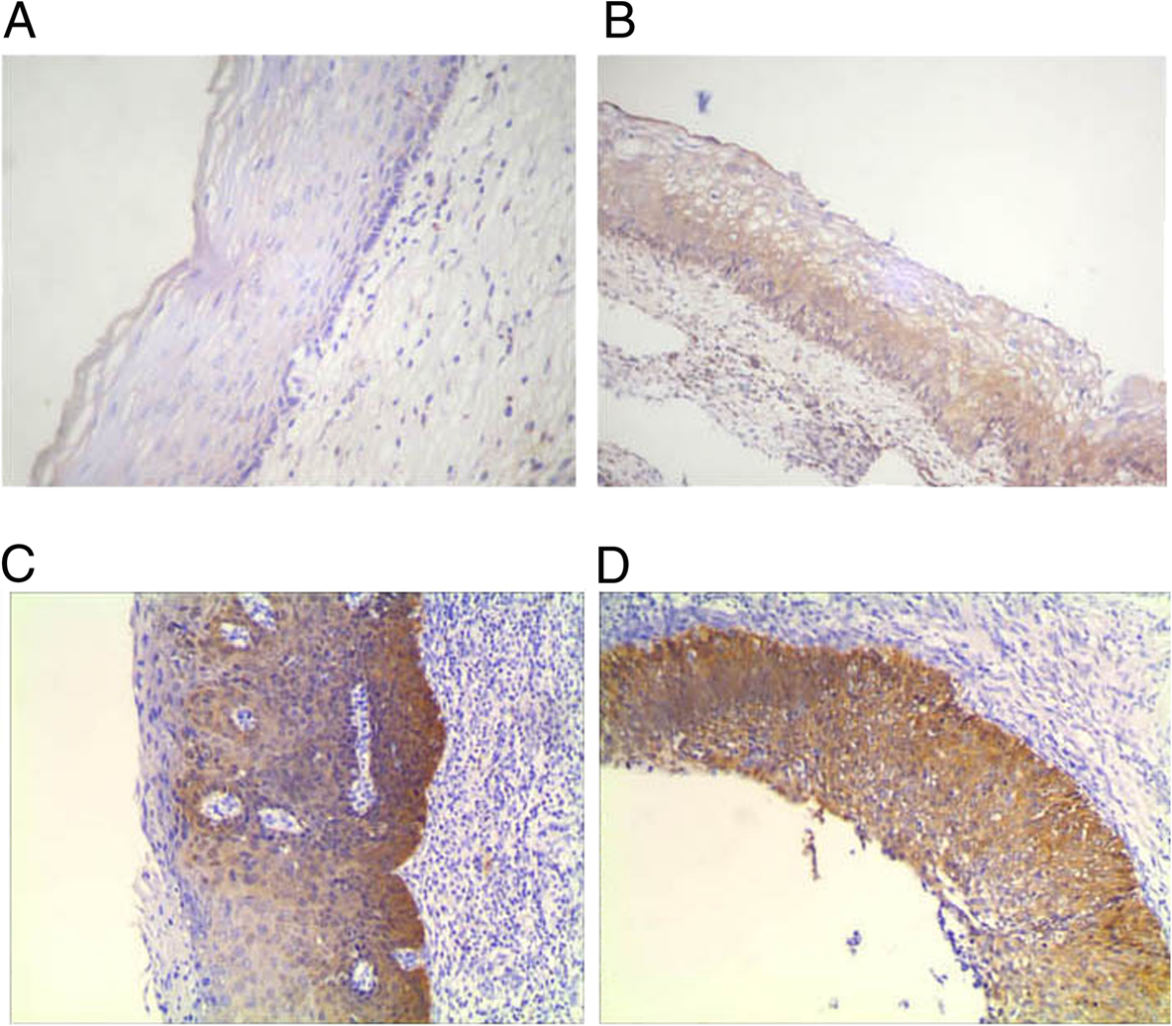
**Immunohistochemical analysis of AEG-1 in normal cervical squamous epithelium (A), CIN I (B), CIN II (C), and CIN III (D).** No AEG-1 staining was detected in the normal cervical squamous epithelium. Immunoreactivity of AEG-1 gradually increased from CIN I, CIN II, to CIN III.

Figure 1 show AEG-1 expression is related to the grade of the lesion in CIN. Expression of AEG-1 was not detected in normal squamous epithelium. Of the 50 CIN lesions, 11 of 15 (73.3%) CIN III were AEG-1 positive, in contrast with only 16.7% (3/18) and 35.3% (6/17) in CIN I and CIN II, respectively. Therefore, a close relationship was observed between the increasing grade of lesion and the intensity of AEG-1 staining in CIN.

### Expression patterns of AEG-1 in cervical cancer

**Figure 2:**
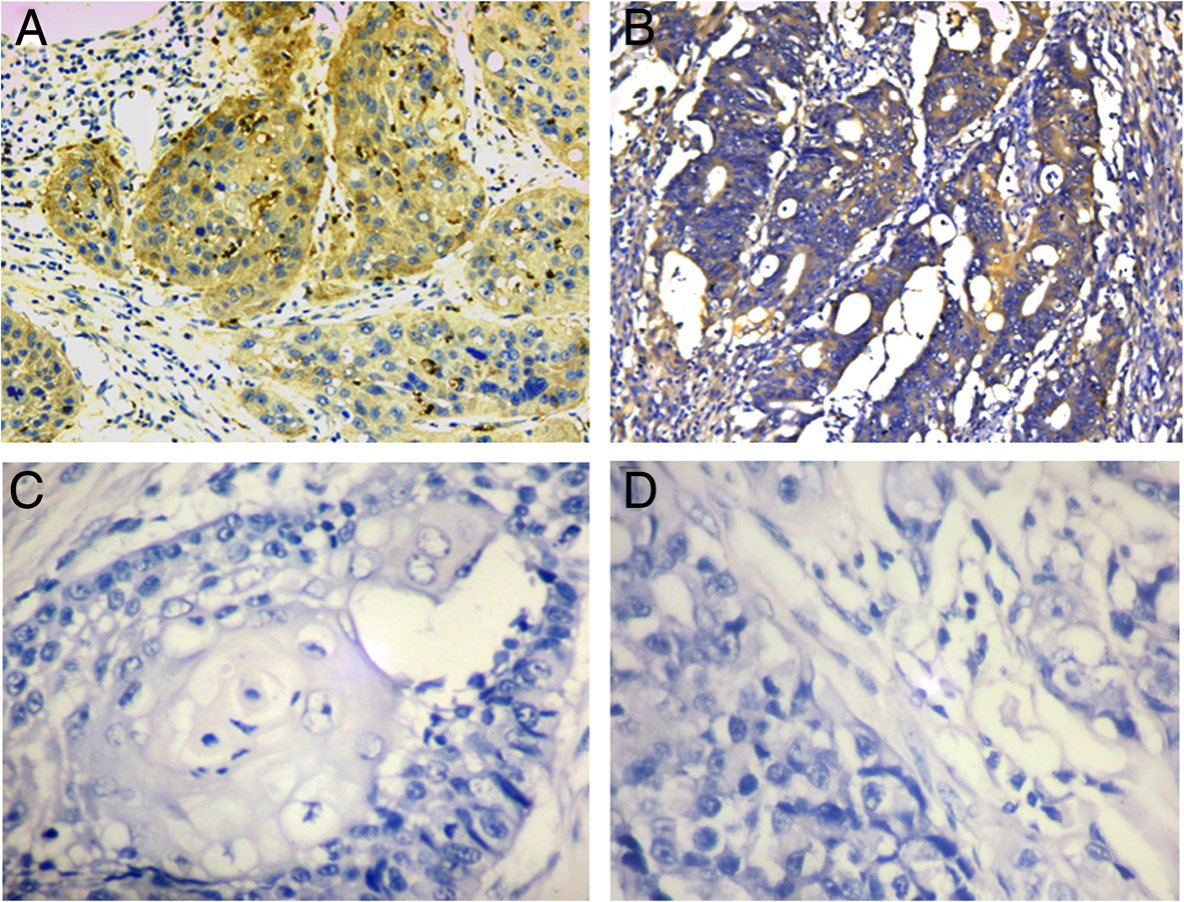
**The expression of AEG-1 in cervical cancer.** Positive expression of AEG-1 in squamous carcinoma **(A)** and adenocarcinoma **(B)**. **(C, D)** Negative controls (200×).

**Figure 3:**
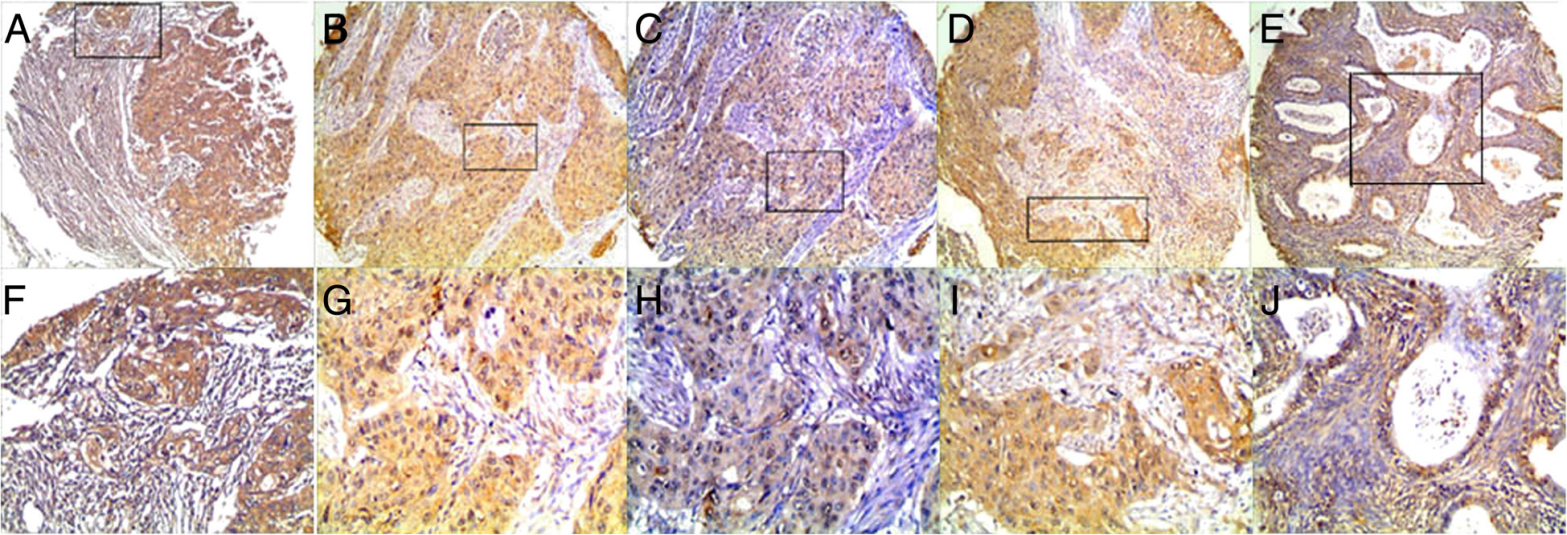
**Immunohistochemistry of AEG-1 in cervical cancer tissue microarray (TMA).** High expression of AEG-1 was observed in squamous carcinoma **(A, B, C, D)** and adenocarcinoma **(E)** (100×). The lower panels indicated the higher magnification (200×) from the area of the box in the upper panels **(F, G, H, I, J)**.

For AEG-1 IHC staining in cervical cancer, immunoreactivity was primarily observed in the cytoplasm of tumor cells (Figures 2 and 3). AEG-1 expression was evaluated informatively in 90 cervical cancers by the TMA constructed previously. The non-informative five TMA samples included samples with too few tumor cells (<300 cells per case) and was replaced by using whole tissue slides. According to ROC curve analysis, expression percentage for AEG-1 above the cutoff value, 40% was regarded as high expression while below the cutoff value was considered as low expression. In the present study, high expression of AEG-1 could be detected in 61.1% (55/90) of cervical cancers. Thirty-five of the 90 (38.9%) cervical cancer samples showed low staining of AEG-1. As shown in Figures 2 and 3, AEG-1 immunoreactivity was predominantly expressed in the cytoplasm of tumor cells. Furthermore, our results suggest that AEG-1 staining from normal cervical specimens, CINs, to cervical cancer was enhanced gradually.

### Selection of cutoff scores for high AEG-1 expression

**Figure 4:**
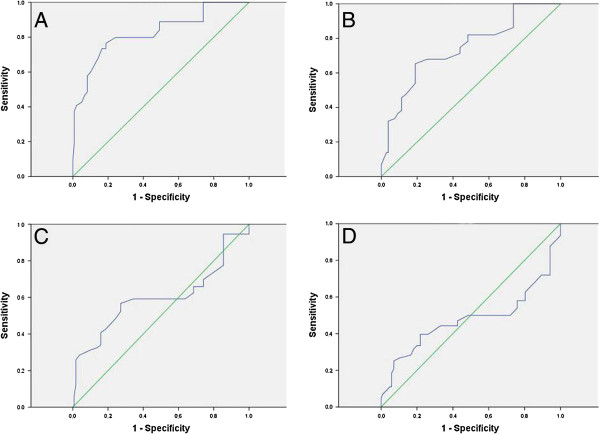
**Receiver operating characteristic curve analysis was employed to determine the cutoff score for the high expression of AEG-1.** The sensitivity and specificity for each outcome were plotted: **(A)** Tumor size, **(B)** lymph node metastasis, **(C)** pathological grade, **(D)** clinical stage.

To identify an optimal cutoff value for high expression, ROC curve analysis was used to determine the cutoff score for expression of AEG-1 in various patterns. The ROC for each clinicopathological parameter (Figure 4) clearly show the point on the curve closest to (0.0, 1.0) which maximizes both sensitivity and specificity for the outcome. Tumors with scores above the obtained cutoff value were considered as high expression of AEG-1 protein leading to the greatest number of tumors correctly classified as having or not having the clinical outcome. The corresponding area under the curve (AUC) and cutoff scores were gathered and shown in Figure 4 and Table 2, respectively. In the present study, tumor size had the shortest distance from the curve to the point (0.0, 1.0), and we selected the cutoff value determined by tumor size. Therefore, the cutoff score for high expression of AEG-1 was defined when >40% of cancer cells had positive staining of AEG-1.

**Table 2:** Area under the curve (AUC) of receiver operating characteristic curve for each clinicopathologic feature

**Variable**	**AUC (95%CI)**	***P*** **value**
Tumor size	0.826 (0.811-0.842)	0.000
Pathological grade	0.598 (0.578-0.619)	0.002
Clinical stage	0.478 (0.456-0.501)	0.046
Lymph node metastasis	0.745 (0.728-0.763)	0.000

### Increased AEG-1 expression correlates with clinicopathologic features of cervical cancer

The high expression rates of AEG-1 in cervical cancer with respect to several clinicopathologic features are presented in previous table. The high AEG-1 expression was significantly correlated with larger tumor size (*P* = 0.010) and lymph node metastasis (*P* = 0.004). There was no significant correlation between AEG-1 expression and other clinicopathologic features, such as patient age, pathological grade, and clinical stage.

### High AEG-1 expression is associated with poor prognosis of patients with cervical cancer

Finally, we detected the value of AEG-1 expression as a predictor of disease outcome in patients with cervical cancer. Assessment of survival in total cervical cancer patients revealed that high expression of AEG-1 was correlated with poor prognosis compared with low cases. Univariate Cox regression analysis suggested that AEG-1 expression, clinical stage, and lymph node metastasis were significantly associated with overall survival. Since features observed to have a prognostic influence by univariate analysis may covariate, AEG-1 expression and those clinicopathologic variables that were significant in univariate analysis were further examined in multivariate analysis (Table 3). Results indicated that high AEG-1 expression was an independent prognostic factor for poor patient overall survival (hazard ratio, 4.021; 95%CI, 1.734-8.283, *P* = 0.027; Table 3). Clinical stage and lymph node metastasis proved to be independent predictors of patient survival (both *P* <0.05). Of all clinicopathologic features, clinical stage was the most independent parameter predicting prognosis (*P* = 0.018).

**Table 3:** Univariate and multivariate analysis of overall survival in patients with cervical cancer

**Factor**	**Univariate analysis**			**Multivariate analysis**		
	**Hazard ratio**	**95%CI**	***P*** **value**	**Hazard ratio**	**95%CI**	***P*** **value**
Age (years)	1.021	0.981-1.052	0.543			
Tumor size	1.678	0.872-3.626	0.257			
Histological classification	0.874	0.258-2.962	0.870			
Clinical stage	6.783	2.345-18.926	0.001	4.677	1. 535–9.794	0.018
Pathological grade	1.467	0.791-3.345	0.342			
Lymph node metastasis	0.108	0.024-0.542	0.002	0.181	0.032-0.642	0.019
AEG-1 high expression	5.894	2.590-18.268	0.004	4.021	1.734-8.283	0.027

**Figure 5:**
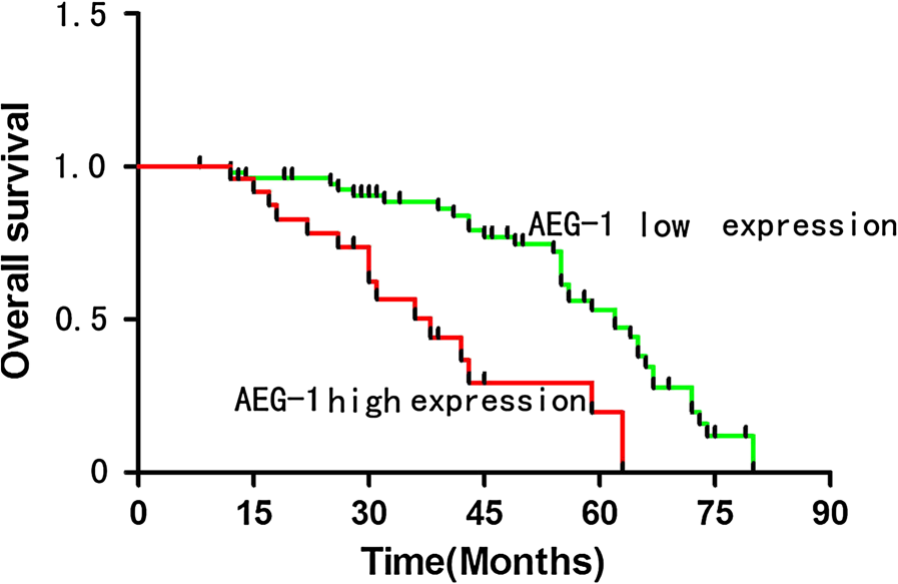
**Kaplan-Meier analysis of overall survival curves of cervical cancer patients according to AEG-1 expression.** The cervical cancer patients with high expression of AEG-1 showed significantly lower overall survival rate than those with low expression of AEG-1.

To elucidate the prognostic role of AEG-1 in cervical cancer patients, overall survival rates was estimated by Kaplan-Meier survival curves. Patients with high expression of AEG-1 had shorter overall survival period than those with low expression (*P* <0.05) (Figure 5).

## Discussion

Recent studies have indicated overexpression of AEG-1 as an important event in a series of cancer. Aberrant AEG-1 expression has been found in some solid tumors including prostateand hepatocellular carcinoma [15,16]. Additionally, AEG-1 expression is increased with the clinical staging of neuroblastoma [17]. Furthermore, AEG-1 expression may represent an independent biomarker for the prediction of prognosis of neuroblastoma and salivary gland carcinoma [18].

Although the important roles of AEG-1 in tumor progression in multiple cancers are now being clarified, its function in development of cervical cancer remains unclear. In the present study, we evaluated the possibility of AEG-1 as a therapeutic target of cervical cancer. We presented the first evidence of AEG-1 upregulation from CIN I to CIN III. Moreover, AEG-1 protein was observed in 61.1% of cervical cancer samples, and AEG-1 expression was found to be significantly correlated with tumor size and lymph node metastasis, as well as unfavorable prognosis of cervical cancer patients. Similarly, Zhang et al. found that 180 of 200 (90%) cervical carcinoma tissue specimens exhibited positive staining for AEG-1 and the upregulation of AEG-1 was significantly correlated with the clinical staging of the patients [19]. These data indicated the important role of AEG-1 in the progression of cervical cancer.

Overexpression of AEG-1 was considered to markedly promote proliferation and tumorigenicity in breast cancer cells, however, an AEG-1-knockdown cell model inhibited cell proliferation and the colony-forming ability [10]. AEG-1 was found to promote the proliferation of various types of cancer cells by activating the PI3K/AKT and NF-κB signaling pathways [20]. Moreover, it was reported that AEG-1 promotes degradation BCCIPα [21]. BCCIPαwas a tumor suppressor and could inhibit growth of cancer cells. Our result also demonstrated upregulation of AEG-1 was correlated with larger tumor size and validated that AEG-1 maybe participated in proliferation of cancer cells.

The metastasis-promoting role of AEG-1 has been confirmed using xenograft model of breast cancer metastasis. In this model, AEG-1 was found to not only promote lung metastasis, but also increase bone metastasis. Conversely, siRNA-silencing of AEG-1 efficiently reduced lung metastasis of cancer cells [22]. Our findings revealed AEG-1 overexpression was associated with lymph node metastasis. As mentioned above, the PI3K/AKT and NF-κB or other signaling pathways maybe involved in lymph node metastasis. However, the molecular mechanism of AEG-1 promotes lymph node metastasis of cancer cells remains unknown and need further elucidated. Furthermore, we found AEG-1 overexpression was correlated with poor prognosis and reduced survival of patients with cervical cancer. Multivariate analysis showed that AEG-1 could be used as an independent prognostic predictor for cervical cancer patients.

## Conclusions

In conclusion, a key finding of the present study is that up regulation of AEG-1 is correlated with tumor growth and lymph node metastasis. Moreover, AEG-1 staining is related to the increasing grade of the lesion in CIN. Although further studies are needed to clarify the role and mechanism of AEG-1 upregulation in the progression of cervical cancer, the present study will provide new insights into the progression of cervical cancers.

### Consent

Consent was obtained from the patient for publication of this paper.

## Abbreviations

AEG-1: High expression of astrocyte elevated gene-1; CIN: Cervical intraepithelial neoplasia; HPV: High-risk human papillomavirus; IHC: Immunohistochemistry; ROC: Receiver operating characteristic; TMA: Tissue microarrays.

## Competing interests

There no conflicts of interest to declare.

## Authors’ contributions

KH performed all the experiments and drafted the manuscript. LL analyzed the clinicopathological data. YM designed the study and was involved in the critical revision of the manuscript. YY contributed to the data analysis. XF participated in data analysis. LS collected the clinical data and coordinated the study. All authors have read and approved the final version of the manuscript.
